# Punishments Enhance Reward Learning by Modulating Striatal Prediction Errors

**DOI:** 10.1523/JNEUROSCI.1631-25.2026

**Published:** 2026-02-24

**Authors:** Joana Carvalheiro, Filippo Queirazza, Lotta Pesonen, Jose Baldaque, Feifan Yao, Rui Zheng, Marios G. Philiastides

**Affiliations:** ^1^School of Psychology & Neuroscience, University of Glasgow, Glasgow G12 8QB, United Kingdom; ^2^School of Health & Wellbeing, University of Glasgow, Glasgow G11 6EW, United Kingdom; ^3^Central European University, Vienna 1100, Austria; ^4^Department of Imaging Neuroscience, UCL Queen Square Institute of Neurology, University College London, London WC1N 3BG, United Kingdom

**Keywords:** fMRI, meta-analysis, punishment, reinforcement learning, reward, striatum

## Abstract

People often make decisions in contexts where rewards and punishments co-occur, yet most human research still examines reward and punishment learning as independent processes. Here, across three studies, we address this gap by demonstrating that punishments amplify reward learning and its neural correlates in healthy human participants. In Study 1 (*N* = 102, 69 females and 33 males), participants performed a probabilistic learning task involving monetary rewards and punishments presented in either intermixed or separated contexts. In intermixed contexts, punishments enhanced reward learning accompanied by changes in computational parameters, including higher learning rates from reward prediction errors. In Study 2 (*N* = 26, 18 females and 8 males), fMRI revealed that punishments amplified reward prediction errors signals in the caudate. Study 3, an fMRI meta-analysis, confirmed that striatal reward responses are consistently stronger when punishments are present. Across studies, we found no reciprocal enhancement of punishment learning by rewards. Together, these findings demonstrate that punishments sharpen reward learning through striatal modulation and underscore the extent to which reward learning is influenced by its broader outcome context.

## Significance Statement

A central question in cognitive neuroscience is how people learn to pursue rewards and avoid punishments. In everyday life, these processes rarely operate in isolation; they often co-occur and interact. For example, a win often feels more rewarding after a loss. Yet, human research has largely examined rewards and punishments separately, overlooking their interplay. Across three studies, we find that punishments enhance reward learning and its underlying striatal prediction-error signals, whereas rewards do not exert a comparable influence on punishment learning. This directional modulation shows that reward learning depends on its broader outcome context, with punishments acting as a key contextual factor. This work offers a theoretical framework for understanding punishment–reward interactions and a new benchmark for future research.

## Introduction

Adaptive behavior depends on learning to seek rewards and avoid punishments, yet it remains unclear whether—and how—these processes influence one another. Everyday experiences suggest an interplay: a win feels stronger after a loss or a warm bath more comforting after cold rain. Correspondingly, empirical human studies showed that painful stimuli can enhance motivation for rewards and modulate reward-related brain responses (Gandhi et al., 2013; Wang et al., 2019). Yet, the learning mechanisms underlying this “cross-talk” remain poorly understood, with studies often using different outcome modalities (e.g., shocks vs money), further hindering direct tests of their interaction.

Nonhuman animal research has long demonstrated interactions between reward and punishment systems. Conditioned suppression studies show that reward-seeking behavior suppressed by punishment cues often rebounds once punishment ends, suggesting compensatory reward pursuit (Estes and Skinner, 1941; LaBarbera and Caul, 1976) possibly through shared or interacting neural pathways (Matsumoto et al., 2016; Schultz, 2016). Despite this body of animal work, most human studies still examine reward and punishment learning separately, even when both occur in the same context (Pessiglione et al., 2006; Sands et al., 2023; Langley et al., 2025), overlooking the possibility that one system may influence the other.

Reinforcement-learning tasks with symmetrical monetary rewards and punishments (i.e., wins and losses) allow direct tests of these effects. Behaviorally, people often learn better from rewards than punishments when both arise from independent choices within the same context (Lin et al., 2020; Carvalheiro et al., 2021a,b; Hao et al., 2023). Interestingly, this asymmetry diminishes when rewards and punishments are learned in separate contexts (Carvalheiro and Philiastides, 2023; Westwood and Philiastides, 2024). These findings raise critical questions about whether punishments can enhance reward learning, whether rewards can likewise improve punishment avoidance learning, and what neurocomputational mechanisms underlie these processes.

We examine these questions within a framework where dopaminergic prediction errors (PEs)—differences between expected and actual outcomes—drive learning (McClure et al., 2003; O’Doherty et al., 2003; Pessiglione et al., 2006). Dopamine encodes reward PEs (Schultz et al., 1997), and although less consistently, punishments can also elicit PE-like dopamine signals depending on modality (Fiorillo et al., 2013; Schultz, 2016) and context (Matsumoto et al., 2016). This suggests that reward and punishment learning may be linked through dopaminergic PE pathways. Punishments may transiently suppress tonic dopamine, increasing the dynamic range of phasic reward-PEs (Tobler et al., 2005; Leknes and Tracey, 2008; Gee et al., 2020), or they may elevate arousal and attention, broadly enhancing PE signals (Aston-Jones and Cohen, 2005). In either case, punishments could serve as contextual amplifiers of reward PEs.

The contextual effects we examine here align with prospect theory and adaptive or relative coding accounts, where outcomes are evaluated against a shifting reference point (Vlaev et al., 2011; Palminteri and Lebreton, 2021). Punishments may lower this reference point, increasing the impact of subsequent rewards. This rationale motivates our approach: we compare reward learning in contexts that include punishments versus those that do not, rather than examining situations in which a single choice can yield either a reward or a punishment as alternative and potentially interacting outcomes. Although economic models often place rewards and punishments on a common scale, computational and neural evidence indicates they are not fully symmetrical (Palminteri and Pessiglione, 2017; Carvalheiro and Philiastides, 2023). We therefore isolate each learning stream and test how context—the presence or absence of punishments—modulates reward learning.

Using behavioral, computational, fMRI, and meta-analytic methods, we show that punishments enhanced reward learning (Study 1) and amplified reward-PE signals in the caudate (Study 2). A meta-analysis further confirmed stronger striatal reward responses when punishments were present (Study 3). We found no evidence that rewards similarly enhance punishment learning. Together, these results show that punishments enhance reward learning by amplifying striatal PE signals when both outcomes are experienced in the same context.

## Materials and Methods

### Study 1: behavioral study

#### Participants

For the behavioral study, data from 102 participants (69 women; age 19–40 years, *M* = 24.79, SD = 3.92) were analyzed. Sample size was determined in G*Power from pilot data (*N* = 17, *d* = 0.28, *α* = 0.05, power = 0.8; two-tailed paired *t* test), indicating a minimum of 104 participants. To allow for exclusions, 121 were recruited from the University of Glasgow and local community. Five were excluded for technical problems and 14 for poor learning, based on prespecified criteria: below-chance accuracy across all six blocks or <40% accuracy in the first 10 trials of each block.

Both studies were approved by the College of Medical, Veterinary & Life Sciences Ethics Committee at the University of Glasgow (200230182). All participants gave informed consent.

#### Probabilistic reinforcement-learning task

Participants completed six blocks of 40 trials each (240 trials in total). The task was adapted from a previous well-established reinforcement-learning paradigm (Pessiglione et al., 2006) and included three types of blocks: (1) rewards-only, (2) punishments-only, and (3) mixed rewards and punishments. Each block type was presented twice, in alternating order and counterbalanced across participants. Blocks were separated by short breaks, and participants were informed of the block type at the start of each block. Prior to the main task, participants completed 12 practice trials for each block type.

Each block contained two pairs of abstract symbols, with 20 trials per pair, randomly selected from a pool of 36 unique symbols presented in a pseudorandomized order, such that the same pair was never presented more than four times consecutively. Each choice pair and context featured different symbols from this pool to promote new learning in every block. Participants made choices by pressing a left or right button and received feedback based on the block type.

In rewards-only blocks, one symbol in each pair yielded a win (+1 point) with probability 0.75 and no outcome (0 points) with probability 0.25, whereas the other symbol had the reverse probabilities (0.25 win, 0.75 no outcome). Thus, each reward-only block comprised two independent stimulus pairs, each with its own probability structure, and participants were instructed to maximize their wins across both pairs.

In punishments-only blocks, the same probability structure applied, but outcomes were either losses or no outcome: one symbol resulted in a loss (−1 point) with probability 0.75 and no outcome with probability 0.25, while the other symbol yielded a loss with probability 0.25 and no outcome with probability 0.75. Thus, each punishment-only block likewise comprised two independent stimulus pairs, each with its own probability structure, and participants were instructed to minimize their losses across both pairs.

In mixed blocks, one pair followed the reward probability structure, while the other followed the punishment probability structure, requiring participants to both maximize wins and minimize losses for each independent pair.

Trials in which participants failed to respond within 1.25 s were followed by a “too slow” message and excluded from further analysis (1.19% of trials).

Participants were informed that all outcomes contributed to their final payment: each win added one point, each loss subtracted one point, and no outcome (“nothing”) left the total points unchanged (zero points). After task completion, all points were summed across trials (summing wins and subtracting losses), and the resulting total was averaged across the six blocks. This final score was then directly converted to the monetary bonus, up to a maximum of £15 for task performance, in addition to a fixed participation payment of £5. If the final score resulted in a negative value, participants still received the full fixed participation payment.

#### Behavioral analysis

Task performance was analyzed with a repeated-measures ANOVA on accuracy with within-subject factors context (mixed vs only) and valence (reward vs punishment), followed by paired *t* tests to describe the interactions. These paired *t* tests were Bonferroni-corrected for multiple comparisons to control family-wise Type I error.

To further examine whether including the interaction term improved model fit, we fit two generalized linear mixed-effects models (GLME) to trial-by-trial individual choice data, Y, coded as correct (1) or incorrect (0), with a binomial distribution and logit link. The first model included context (mixed-reward and punishment or only-reward and punishment), valence (reward or punishment), block number (1 or 2), and trial number as fixed effects (Eq. 1), while the second model additionally included the context × valence interaction term (Eq. 2). Both models included random intercepts for subjects and blocks. To account for individual variability in learning rates, we also included random slopes for trials within subjects. A likelihood ratio test was conducted to compare the models with and without the interaction term:

$$\mathrm{logit}\;(P(Y_{\mathrm{ijkt}}=1))=\;\beta_{0}+\;\beta_{1}\mathrm{Contex}t_{k}+\beta_{2}\mathrm{Valenc}e_{k}+\;\beta_{3}\mathrm{Bloc}k_{j}+\beta_{4}\mathrm{Tria}l_{t}+(1+\mathrm{Tria}l_{t}|\mathrm{Subjec}t_{i})+(1|\mathrm{Bloc}k_{j}),\;(1)$$

$$\mathrm{logit}\;(P(Y_{\mathrm{ijkt}}=1))=\;\beta_{0}+\;\beta_{1}\mathrm{Contex}t_{k}+\beta_{2}\mathrm{Valenc}e_{k}+\;\beta_{3}\mathrm{Bloc}k_{j}+\beta_{4}\mathrm{Tria}l_{t}+\beta_{5}(\mathrm{Contex}t_{k}\times \mathrm{Valenc}e_{k})+(1+\mathrm{Tria}l_{t}|\mathrm{Subjec}t_{i})+(1|\mathrm{Bloc}k_{j}),\;(2)$$

where i indexes participants, j blocks, k experimental conditions (context and valence), and t trials.

Stay/switch behavior was analyzed by comparing the proportion of repeated choices after positive feedback (reward or avoided loss) to the proportion of switches after negative feedback (no reward or loss) via repeated-measures ANOVA, with paired *t* tests for significant effects.

Choice latency was analyzed with a repeated-measures ANOVA on average response times with within-subject factors context (mixed vs only) and valence (reward vs punishment), followed by planned paired *t* tests.

Effect sizes are reported as *η*^2^ for ANOVAs and Cohen's *d* for post hoc *t* tests.

#### Reinforcement-learning model

A standard reinforcement-learning algorithm was used to estimate learning rates based on each participant's trial-by-trial choices and feedback.

Initial *Q* values were set to −0.5 for punishment pairs and 0.5 for reward pairs. This provided a better fit to the behavioral data than initializing at 0 in the mixed context, where possible outcomes were −1, 0, and 1. Allowing the initial *Q* value to vary also fitted the behavioral data well, but Bayesian Information Criterion (BIC) showed no improvement over the fixed *Q* model, so the extra parameter was omitted. Across all three initialization schemes, the main effect of context on reward-learning rates remained significant (all *p* < 0.034).

For each trial, t, within a pair of stimuli, the value of the chosen stimulus (say *A* was chosen) was updated according to the following:

$$Q_{A}\;(t+1)=Q_{A}(t)+\alpha \;*\;\delta (t),\;(3)$$

where δ was the prediction error (PE):

$$\delta (t)=r(t)-Q_{A\;}(t),\;(4)$$

where r(t) was 1 for wins and 0 for neutral outcomes in reward pairs and −1 for losses and 0 for neutral outcomes in punishment pairs. Under this outcome coding, positive and negative PEs corresponded to reward delivery versus omission in reward pairs and to punishment omission versus delivery in punishment pairs. Accordingly, separate learning rates were estimated for positive and negative PEs in reward and punishment pairs. The learning rate, α, was given by the following:

$$\alpha =\{\begin{array}{cc} \alpha^{+}, & \mathrm{if}\;\delta (t)>0\\ \alpha^{-}, & \mathrm{if}\;\delta (t)<0 \end{array},\;(5)$$

where $\alpha^{+}$ and $\alpha^{-}$ were the learning rates for positive and negative PEs, respectively.

The probability of choosing one stimulus over another (say *A* over *B*) was given by the *softmax* equation:

$$P_{A}(t)=\;\frac{e^{\left[Q_{A}(t)\;*\;\beta \right]}}{e^{\left[Q_{A}(t)\;*\;\beta \right]}+\;e^{[Q_{B}(t)\;*\;\beta ]\;}},\;(6)$$

where the *β* parameter, or inverse temperature, reflects the noise in choice selection. *β* was estimated as a free parameter to account for individual differences in choice consistency and to account for the coupling of learning rate estimates with decision noise. Still, we confirmed that fixing *β* at 10 did not change the interpretation of our key result on the reward-learning rates.

Model fitting involved estimating the free parameters (*α^+^*, *α^−^*, and *β*) that best captured trial-by-trial choices in each context, using maximum likelihood estimation (MLE) and a constrained nonlinear optimization procedure (implemented via *fmincon* in MATLAB), separately for each participant. We opted for MLE over maximum a posteriori (MAP) estimation to avoid imposing prior assumptions across the two datasets, which differed slightly in task structure (Study 1: 0.75/0.25 outcome probabilities; Study 2: 0.7/0.3, slightly more challenging as indicated by higher exclusion rates). For completeness, we also fitted the model using MAP with weakly informative priors—Beta (1.1, 1.1) for *α^+^* and *α^−^*; Gamma (15, 0.75) for *β*—which produced qualitatively similar results. PEs from MLE and MAP were highly correlated across all four conditions (*r* > 0.99), demonstrating that our modeling and model-based fMRI findings were robust to the fitting approach.

Goodness of fit was assessed by comparing predicted choice probabilities (*softmax* output, Eq. 6) to observed choices across trials (learning curves) in Study 1. Parameter recovery was evaluated by simulating task-choice behavior (given the stimulus-outcome probabilities) for 102 virtual participants based on their estimated parameters in each condition, running 500 simulations. We averaged correct choices in the simulated data to confirm that they followed the same pattern as the observed data. Then, for each simulation, we fitted the model to the virtual participants’ data to estimate new (recovered) parameters and tested their correlation with the original parameters using Pearson's correlations.

We also tested a model that included an additional reward-sensitivity parameter (*ρ*), which scaled outcome magnitudes before updating the value of the chosen option. This parameter was introduced to account for the possibility that individual differences in outcome sensitivity, rather than learning rate, might drive variation in choice behavior. The extended model therefore added a fourth free parameter, *ρ*, such that the PE was computed as follows:

$$\delta (t)=\rho .r(t)-Q_{A\;}(t).\;(7)$$

Fitting and parameter recovery proceeded identically to the main model described above. However, the reward-sensitivity parameter showed very poor identifiability, with strong collinearity between other parameters and did not improve model fit. Likelihood ratio tests comparing the nested three-parameter (no reward sensitivity) and four-parameter (with reward sensitivity) models, together with BIC values, confirmed that this extended model did not provide additional explanatory power. Given these issues, and to maintain interpretability, all reported analyses used the simpler model (Eqs. 3–6).

After validating the model, we performed separate repeated-measures ANOVAs on the estimated learning rates (*α*^+^ and *α*^−^), with context and PE valence as within-subject factors, followed by post hoc paired *t* tests. This analysis directly tested our primary hypothesis regarding context-dependent differences in learning rates. Given that *α*^±^ and β are often inversely coupled (Fig. S2*B*), and their product is more reliably estimated than individual parameters with larger errors (Schönberg et al., 2007), we additionally performed repeated-measures ANOVAs on the product of learning rates and inverse temperature (*α*^±^ × *β*), as a complementary control analysis.

Effect sizes are reported as *η*^2^ for ANOVAs and Cohen's *d* for post hoc *t* tests.

To assess whether behavioral differences between contexts were better explained by changes in *α* and/or *β*, we fit four variants of the reinforcement-learning model (estimated separately for reward and punishment trials). For simplicity, each variant used a single learning rate (*α*), rather than separate learning rates for positive (*α*^+^) and negative (*α*^−^) PEs. The null model assumed shared *α* and *β* across mixed and separate contexts. The *β*-only model allowed *β* to vary by context while keeping *α* fixed. The *α*-only model allowed *α* to vary by context while keeping *β* fixed. The full model allowed both parameters to vary by context. Model fits were compared using BIC. We then directly compared the *α*-only and *β*-only models, which have the same number of parameters and directly test our hypothesis of context-dependent changes in *α* versus *β*, using random-effects Bayesian model comparison as implemented in the Variational Bayesian Analysis (VBA) toolbox (Daunizeau et al., 2014). For the VBA procedure, we supplied the per-subject log model evidence (approximated by BIC) for each reward and punishment condition for both models (*α*-only and *β*-only). For each model, we also simulated 500 datasets using the fitted parameters and compared them against the observed data.

To further dissociate the contributions of *α* and *β* to reward performance, we ran linear regressions with mean accuracy as the dependent variable and the estimated parameters from our main model (*α*^+^, *α*^−^, *β*) as predictors, separately for the reward-mixed and reward-only contexts.

### Study 2: fMRI study

#### Participants

For the fMRI study, data from 26 participants (18 women; age 19–42 years, *M *= 25.73, SD = 6.00) were analyzed. Power analysis (effect size from Study 1: *d* = 0.47, *α* = 0.05, power = 0.8; one-tailed paired *t* test) indicated a minimum of 30 participants. A one-tailed test was used to calculate the sample size because Study 2 was designed a priori as a confirmatory replication of the directional effect observed in Study 1—higher accuracy for rewards than punishments in mixed relative to separate contexts. To allow for exclusions, we tested 36 participants. Three were excluded for technical problems or incidental findings, and seven for poor learning (same criteria as Study 1).

#### Probabilistic reinforcement-learning task

The task was identical to Study 1 except for the following (1) outcome probabilities of 0.70/0.30 instead of 0.75/0.25 and (2) jittered interstimulus and intertrial intervals (1–4 vs 1–1.5 s in Study 1).

Participants received £10 plus up to £15 based on performance (calculated as in Study 1).

#### Behavioral analysis and reinforcement-learning model

We conducted targeted one-tailed *t* tests on the key directional contrasts from Study 1, including the interaction used for the sample size calculation (reward-mixed − punishment-mixed) > (reward-only − punishment-only) to evaluate replication of the behavioral effects. Behavioral post hoc contrasts were Bonferroni-corrected to control family-wise Type I error.

The reinforcement-learning model and the statistical analyses of its estimated parameters were conducted using the same procedures as described in Study 1.

Effect sizes are reported as *η*^2^ for ANOVAs and Cohen's *d* for post hoc *t* tests.

#### fMRI data acquisition and preprocessing

Functional and structural MRI data were acquired using a Siemens 3 Tesla TIM Trio MRI scanner with a 32-channel head coil. A 5.5 min T1-weighted anatomical scan was obtained (isotropic voxel size: 1 mm), along with multislice T2-weighted echoplanar images (EPIs) with BOLD contrast. Each EPI volume comprised 68 slices acquired in an interleaved-ascending order with an isotropic resolution of 2 mm. Imaging parameters were as follows: repetition time (TR), 2,000 ms; echo time (TE), 26 ms; flip angle, 60°; field of view, 192 × 192 mm; matrix, 96 × 96 mm.

Before preprocessing, data from the two blocks of each context (reward-only, punishment-only, and mixed) were concatenated. Reward-only and punishment-only datasets were kept fully independent (as in the experimental task) to preserve valence specificity and avoid cross-contamination of reward and punishment signals.

In mixed blocks, reward and punishment trials were temporally interleaved, creating potential hemodynamic overlap and shared variance, particularly between outcome-related activity (our phase of interest) and the subsequent decision phase. To address this, when modeling activity for one valence (e.g., reward trials), all trials of the opposite valence within the same mixed block (e.g., punishment trials) were also concatenated into a single nuisance regressor modeling the decision/choice phase and control for any shared variance (see “fMRI analysis” below). Specifically, this concatenation allowed us to model variance associated with perceptual and motor processes at the decision phase, elicited by both same- and opposite-valence trials, removing their contribution from the regressor of interest. As a result, reward- and punishment-related signals were statistically separated despite temporal interleaving in mixed blocks, reducing cross-valence contamination when estimating effects for either outcome type.

Concatenation was performed in several steps. First, intensity normalization was applied within each block. Next, a linear registration step was conducted by aligning an exemplar EPI from each block to an exemplar EPI from the first block of the same condition. The resulting transformation matrices were then applied to all EPIs, ensuring spatial consistency across blocks. The concatenated datasets consisted of 560 volumes per condition, with separate datasets for reward trials (including both reward-mixed and reward-only trials, with punishment-mixed trials as confounders) and punishment trials (including both punishment-mixed and punishment-only trials, with reward-mixed trials as confounders). Head motion parameters were estimated separately for each block using the MCFLIRT tool (Jenkinson et al., 2002) and subsequently concatenated.

Preprocessing was performed using FSL FEAT (Jenkinson et al., 2012) and included slice-timing correction, skull stripping, high-pass filtering (cutoff, 100 s), and spatial smoothing (6 mm FWHM Gaussian). EPIs were registered to MNI space using FNIRT (Andersson et al., 2007) with a 10 mm warp resolution, via an intermediate transformation to anatomical space (six-parameter rigid body) followed by nonlinear warping.

#### fMRI analysis

To assess context effects on striatal PE signals, we first modeled trial-by-trial signed PEs as continuous parametric modulators of the BOLD response and compared the resulting contrasts (reward-mixed > reward-only and vice versa; punishment-mixed > punishment-only and vice versa). However, because this approach primarily indexes slope differences in the PE–BOLD relationship, it can miss context effects expressed as baseline shifts.

To capture and visualize such effects, signed PEs from the reinforcement-learning model (Eqs. 3–6) were divided into three equally sized bins (low, medium, high). Mean PE magnitudes in these bins did not differ between mixed and separate contexts for either rewards (all *p* > 0.42) or punishments (all *p* > 0.51).

Functional data were analyzed with a general linear model (GLM) in FSL FEAT. At the first level, we estimated simple within-subject activations for each PE bin in reward-mixed and reward-only contexts (used later for region of interest analysis) and contrasts comparing contexts (reward-mixed > reward-only) for each bin (used later for meta-analysis comparisons).

The GLM was time locked to the outcome phase (when PEs are generated). Six boxcar regressors of interest were included, each with a duration of 750 ms to match the presentation of the outcome: three regressors modeled reward-mixed trials for PE bins 1, 2, and 3, while three regressors modeled reward-only trials for the same PE bins. All event amplitudes were set to 1.

Additional nuisance regressors were included in the model to account for potential confounds. These included a regressor for the choice phase (accounting for general decision-related activity such as motor preparation, attention, or perceptual demands), modeled as a boxcar function with trial-wise choice latencies, allowing us to capture sustained activity across the decision period and to account for latency differences between reward and punishment trials. In mixed contexts, this nuisance regressor included choices for both reward and punishment pairs, mitigating confounding activations related to trial-type alternation. We also included nuisance regressors for missed choices and outcomes and regressors marking concatenation points across blocks. Six motion regressors (three translations and three rotations) were also included to correct for motion-related artifacts. A separate first-level GLM for punishment trials followed the same design.

By modeling choice and outcome events using boxcar regressors, we captured sustained BOLD responses across each task period to accommodate variability in response times and phase durations across participants.

To validate comparability with meta-analysis data, we ran a supplementary GLM using unmodulated valence regressors for positive (reward) and negative (no reward) outcomes instead of PE bins. This approach matched the structure of the meta-analytic model, which was based on outcome valence contrasts (e.g., reward > absence of reward), rather than PE bins. This supplementary analysis confirmed that the results remained consistent with those obtained in the main GLM, supporting the robustness and comparability of our findings with the meta-analysis (see below, Study 3: fMRI meta-analysis).

#### Regions of interest analysis

BOLD signal estimates (beta coefficients) for regressors of interest were extracted from three bilateral striatal subregions: nucleus accumbens, caudate, and putamen. Estimates were averaged across all voxels within each region of interest (ROI). ROIs were defined using the Harvard–Oxford Subcortical Structural Atlas in FSL (thresholded at 50% probability) and back-projected into each participant's EPI space using the inverse registration transform.

For the parametric modulation analysis, contrast estimates indexing contextual effects on PEs (reward mixed > reward only and punishment mixed > punishment only) were averaged from each ROI and tested against zero using two-tailed one-sample *t* tests.

For the binned PE analysis, beta estimates for PE bins 1–3 in mixed- and single-outcome contexts were extracted and averaged within each ROI. Repeated-measures ANOVAs tested the effects of context (mixed vs only) and PE bin (1–3) on BOLD estimates. To control family-wise Type I error arising from testing multiple ROIs, *p*-values from these ROI-specific ANOVAs were Bonferroni corrected. In addition, a separate ANOVA including subregion (nucleus accumbens, caudate, putamen) as a within-subject factor assessed regional specificity. Effect sizes are reported as *η*^2^ (ANOVAs) and Cohen's *d* (post hoc *t* tests).

#### fMRI corrections for multiple comparisons

Although the primary analysis focused on striatal ROIs, we conducted exploratory whole-brain analyses to compare our fMRI activations with meta-analytic results.

To control for false positives, 3dClustSim (Cox et al., 2017) was used to establish cluster extent thresholds corrected for multiple comparisons across the whole brain. This method performed 10,000 Monte Carlo simulations, accounting for both brain volume and data smoothing to determine cluster-size thresholds. A voxel-wise threshold of *p* = 0.01 (*Z* = 2.3) was applied, with a cluster-wise probability threshold of *p* < 0.05. Based on 5,000 permutations, 3dClustSim determined that a minimum cluster size of 99 voxels was required for whole-brain results to survive correction. These results were then used to establish statistical significance thresholds for activation clusters and to quantify their overlap with findings from the meta-analysis (see below, Study 3: fMRI meta-analysis).

#### fMRI time series extraction and analysis based on punishment history

To examine how recent punishment history modulates reward-related activity, trial-wise BOLD time series were extracted from three striatal ROIs (nucleus accumbens, caudate, putamen). Subject-specific anatomical masks were defined in functional space and applied to preprocessed, unsmoothed EPI volumes. Within each mask, voxel-wise BOLD signals were averaged for each subject. Event-related time courses were constructed for reward trials in mixed blocks, time locked to outcome onset (when PEs occur). For each trial, we extracted BOLD signal from 2 volumes before to 8 volumes after onset (TR, 2 s) and normalized to a preoutcome baseline (−4 to 0 s) and converted to percent signal change.

To assess the influence of punishment history, reward trials were grouped in two ways. First, by the number of prior punishments within the same block (reset after each reward) into three bins—no prior punishments, one prior punishment, and two or more prior punishments. Second, by punishment recency, dividing trials into three equally sized bins based on time since the most recent punishment (also reset after each reward).

To test for a parametric effect of punishment history, trial-averaged BOLD signal at 6 s postreward (peak of BOLD response) was extracted for each bin, and a linear regression across the three levels was fitted for each subject in MATLAB. Slopes were tested against zero using a one-tailed one-sample *t* test, where a significant positive slope indicated stronger reward responses with increasing punishment history.

A second analysis assessed whether this effect reflected a ramping signal following an initial dip, modulated by punishment recency. For each bin, the difference in signal between 0 and 6 s postreward was calculated, and the same slope-fitting and statistical testing procedure was applied to these ramping estimates.

Finally, to ensure that punishment history effects in the caudate were not confounded by independent learning trajectories within reward and punishment domains, we confirmed that caudate reward responses at 6 s postreward did not differ between the first and second halves of each block (*p* = 0.85) and that the number of prior punishments was not significantly different between block halves (*p* = 0.68). These checks indicate that the observed modulation of reward signals by punishment history is unlikely to reflect learning trajectory differences or temporal drift across trials.

### Study 3: fMRI meta-analysis

#### Literature search and inclusion criteria

Our meta-analysis followed the Preferred Reporting Items for Systematic Reviews and Meta-Analysis (PRISMA) guidelines. We conducted a systematic search in PubMed, using the following search terms in titles or abstracts: (fMRI OR imaging OR neuroimaging) AND ((predict OR prediction OR predictive) AND (error OR errors)) OR (reward OR punishment OR appetitive OR aversive OR reinforcement OR reinforcing) AND (learning OR conditioning). Separate searches were conducted for 1999–2022 and 2022–2025, with publication date restricted to January 2025 at the latest. We applied the additional following filters: humans, english, adult: 19–44 years, adult: 19+ years, young adult: 19–24 years.

We first reviewed abstracts and excluded those that were irrelevant (e.g., animal data only, reviews, etc.). Full-text articles were then read by the first-author (JC) to determine final inclusion. A total of 1,135 abstracts were identified through the database search, with one additional abstract identified through the authors’ sources (totalling 1,136 abtracts). After removing duplicates, 1,124 abstracts were reviewed. Following this screening process, 641 full-text articles were evaluated. Of these, 25 studies met the inclusion criteria, with 16 categorized as reward-mixed and 9 as reward-only, representing a total of 204 coordinate sets, across 706 participants.

Only published, peer-reviewed, original research articles were considered. Included studies met the following criteria: (1) they included human adult participants 18–65 years old; (2) they employed fMRI; (3) they used fMRI analyses that were time locked to the presentation of outcomes (feedback); (4) they used image subtraction (binary contrasts) of a reward regressor (e.g., win) against a neutral regressor (e.g., no-win or nothing) to determine activation foci; continuous parametric analyses of PEs were excluded; (5) they used outcomes consisting of abstract points, monetary payoffs, consumable liquids, and arousing pictures but excluded articles in which outcomes consisted of social or correct/incorrect feedback; (6) they did not use any pharmacological interventions; (7) they did not include genotyping; (8) they reported whole-brain coordinates (i.e., region of interest contrasts were excluded); (9) they provided Talairach or Montreal Neurological Institute (MNI) coordinates. Coordinates reported in Talairach space were converted to MNI space using the Tal2MNI algorithm implemented in MATLAB.

#### Study categorization

The goal of this meta-analysis was to classify studies into two distinct contexts—reward-mixed and reward-only—to examine differences in neural reward representations. The reward-mixed context included learning trials in which both reward and punishment outcomes could occur within the same block. The reward-only context included learning trials in which participants could receive rewards of varying magnitudes or no reward, but no punishments. Studies were categorized based on these definitions, and their fMRI contrasts were reviewed to confirm their classification.

Studies using continuous parametric contrasts of PEs were excluded because our empirical data suggested that differences in PE responses were best captured by a main effect rather than by variations in the slope, ensuring comparability with our dataset. Studies with correct/incorrect feedback were also excluded because their context was ambiguous: incorrect feedback could be interpreted as either a loss or punishment (consistent with the reward-mixed context) or the absence of a reward (consistent with the reward-only context). As a control, we included 10 additional studies using correct/incorrect to test whether their inclusion in either context affected our findings.

Additionally, a search was conducted for studies that specifically examined neutral versus reward contrasts across both contexts. However, only four studies met this criterion (Abler et al., 2005; McGuire et al., 2014; Johnston et al., 2015; Romaniuk et al., 2019), which was insufficient for a minimally informative meta-analysis.

#### Statistical analysis

To quantitatively compare neural reward representations between the reward-mixed and reward-only contexts, a Multi-Level Kernel Density Analysis (MKDA; Wager et al., 2009) was performed. This method accounts for multiple activation foci within individual studies by treating each study as a single unit, reducing bias from studies reporting a high number of activation coordinates. However, because there was an unequal number of studies in each category (16 reward-mixed and 9 reward-only), the analysis still risked being biased toward the larger category.

To address this imbalance, a bootstrapping procedure with 500 iterations was performed. In each iteration, a random subset of 9 studies was selected from the reward-mixed category to match the number of studies in the reward-only category. For each bootstrapped iteration, MKDA maps were generated, and significant clusters of convergence were identified using a Monte Carlo permutation test with 1,000 iterations. Significance thresholds were set at *p* < 0.05 with family-wise error (FWE) correction. The final meta-analytic contrast map for reward-mixed versus reward-only was obtained by averaging across all 500 bootstrapped iterations and applying a 5% consistency threshold to ensure minimal variability across iterations. This approach ensured that any observed differences between reward-mixed and reward-only contexts were not primarily driven by the unequal number of studies.

#### Overlap with empirical data

To assess convergence between the meta-analysis and empirical fMRI results, we examined spatial overlap of significant clusters identified in both approaches. For the meta-analysis, binary activation maps were generated based on the MKDA approach described above. For the empirical fMRI data, significant clusters were identified using a *Z*-value threshold of 2.3 with cluster correction (see above, fMRI Corrections for multiple comparisons). The spatial overlap between the two datasets was visualized across the whole brain. Additionally, the percentage of overlapping voxels within a predefined anatomical mask of the striatum—including the nucleus accumbens, caudate, and putamen—was quantified.

## Results

### Study 1: behavioral dataset

#### Punishments enhance reward-seeking performance

**Figure 1.**
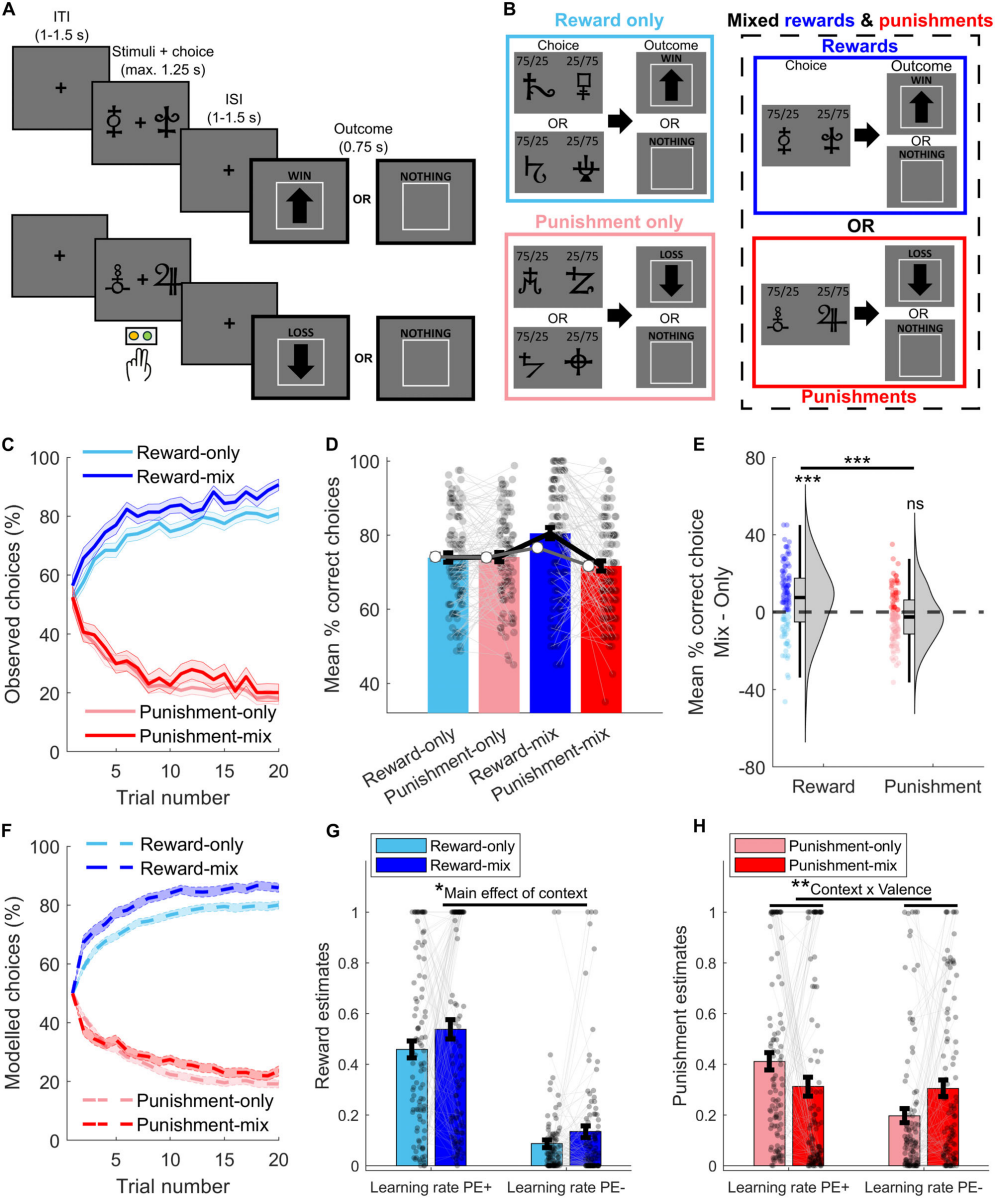
Study 1, Behavioral dataset: Reinforcement-learning task, behavioral performance, and model fitting results (*N* = 102). ***A***, Exemplary trials for reward (top) and punishment (bottom). In the fMRI version, the interstimulus interval (ISI) and intertrial interval (ITI) varied between 1 and 4 s. ***B***, Participants completed a reinforcement-learning task with three context (block) types (counterbalanced and alternated): (1) Reward-only context, where choices led either to a win or nothing; (2) Punishment-only context, where choices led to a loss or nothing; and (3) Mixed context, where one symbol pair led to a win or nothing (reward-mix) and another pair led to a loss or nothing (punishment-mix). On each trial participants selected one of the symbols in a pair, each associated with a 0.75 or 0.25 probability of their respective outcome (adjusted to 0.7/0.3 in the fMRI version). Each participant completed 240 trials, with 20 trials per each pair of symbols (40 trials per context, and each context repeated twice). ***C***, Learning curves show the percentage of participants selecting the “correct” reward stimulus (higher-probability reward, blue) and the “incorrect” punishment stimulus (higher-probability loss, red) across trials. ***D***, Behavioral performance across contexts. Colored bars represent mean accuracy (percentage of choices for the high-probability reward or low-probability punishment stimuli), with black error bars representing ± SEM. White dots depict accuracy estimated from reinforcement-learning model simulations, which closely align with observed data (*p* < 0.001 interaction: mix/separate × reward/punishment). ***E***, Violin plots illustrate differences in performance between mixed and separate contexts for rewards (blue) and punishments (red), showing a larger effect for rewards (*d* = 0.38) and a weaker effect for punishments (*d* = −0.18). ***p < 0.001; ns *p* > 0.05. ***F***, Model-estimated choice probabilities for selecting the “correct” reward (top) and “incorrect” punishment (bottom) symbols, mirroring the observed learning curves in ***C***. ***G***, ***H***, Model-estimated learning rates for positive (PE+) and negative (PE−) prediction errors in reward (***G***) and punishment (***H***) trials across the different contexts. For reward trials, PE^+^ corresponds to better-than-expected rewards (reward delivery), and PE^−^ corresponds to worse-than-expected rewards (reward omission). For punishment trials, PE^+^ corresponds to better-than-expected punishments (punishment omission), and PE^−^ corresponds to worse-than-expected outcomes (punishment delivery). *p < 0.05, main effect of context (Reward-mixed > Reward-only); **p < 0.01, context × valence interaction. Error bars represent SEM; lines connect individual participants.

In a first behavioral study (*N* = 102), we examined how people learned from rewards and punishments when presented separately or within the same context. For this, each participant completed a reinforcement-learning task across three contexts: (1) reward-only, with two choice pairs leading to wins or nothing; (2) punishment-only, with two choice pairs leading to losses or nothing; and (3) mixed rewards and punishments, with one choice pair leading to wins or nothing and another leading to losses or nothing (Fig. 1*A*,*B*).

As expected, participants gradually learned to obtain rewards and avoid losses across all contexts (all learning slopes significantly different from zero, *p* < 0.001; Fig. 1*C*). However, a significant interaction effect between context (mixed vs separate) and valence (reward vs punishment) indicated that learning patterns differed between contexts (*F*_(1,101)_ = 22.75, *p* < 0.001, *η*^2^ = 0.049; Fig. 1*D*). Model comparison using generalized linear mixed-effects models further confirmed that including an interaction term significantly improved model fit (*χ*^2^_(1)_ = 66.92, *p* < 0.001).

Post hoc comparisons showed comparable performance between reward-only and punishment-only contexts (*t*_(101)_ = −0.13, *p* = 0.90, *d* = −0.013). However, in the mixed context, participants were significantly better at seeking rewards than avoiding punishments (*t*_(101)_ = 5.22, *p* < 0.001, *d* = 0.52).

Critically, direct comparisons across contexts revealed that participants were better at seeking rewards in the mixed context—where punishments were present—than in the reward-only context (*t*_(101)_ = 3.78, *p* < 0.001, *d* = 0.38), suggesting that punishments enhance reward learning (Fig. 1*E*). Moreover, this effect was mostly unidirectional: the presence of rewards did not significantly change punishment learning (*t*_(101)_ = −1.86, *p* = 0.066, *d* = −0.18; Fig. 1*D*,*E*). All reported significant effects survived Bonferroni’s correction for multiple comparisons (all Bonferroni-adjusted *p* < 0.001).

Complementary analysis of stay-switch behavior supported these findings: on reward trials, but not punishment trials, the stay-after-positive/switch-after-negative ratio was higher in the mixed than the reward-only context (*t*_(101)_ = 4.34, *p* < 0.001, *d* =0.43; Fig. S1*A*). We also verified that choice latencies for reward trials did not differ significantly between contexts (*t*_(101)_ = 0.28, *p* = 0.78, *d* = 0.03; Fig. S1*B*), indicating that enhanced reward learning in mixed blocks is not explained by slower, more deliberate responding.

To rule out potential confounds from task structure, we confirmed that the observed effects were not driven by asymmetries in choice behavior within the reward-only and punishment-only contexts. In the reward-only and punishment-only contexts, participants learned from two stimulus pairs with identical outcome structures (win/nothing or loss/nothing; Fig. 1*B*). If participants consistently performed better on one pair than the other within these contexts, this could bias their averaged performance. However, accuracy did not differ between the two pairs within either the reward-only or punishment-only context (all *p* > 0.054). We further confirmed the robustness of the effects by randomly sampling performance from only one pair per reward-only and punishment-only contexts across 10,000 iterations (Fig. S1*C*).

Overall, these findings indicate that the presence of punishments enhances reward-seeking behavior, suggesting faster learning from reward outcomes.

To test whether punishments increased reward-learning rates—a mechanism not directly observable from behavior alone—we next applied computational modeling.

#### Punishments enhance reward-prediction-error–based learning processes

To examine the learning mechanisms underlying enhanced reward-seeking behavior in the presence of punishments, we fit a reinforcement-learning model that estimated separate learning rates for positive and negative PEs separately for reward and punishment trials. For rewards, positive PEs reflect better-than-expected rewards (reward delivery) and negative PEs reflect worse-than-expected rewards (reward omission). For punishments, positive PEs reflect better-than-expected punishments (punishment omission) and negative PEs reflect worse-than-expected outcomes (punishment delivery). Distinguishing these learning rates is motivated by robust neurobiological evidence that dopamine neurons differentially encode positive and negative PEs during reward learning, and possibly during punishment learning as well (Matsumoto and Hikosaka, 2009). By modeling these processes separately, we aimed to gain clearer insight into the neurocomputational mechanisms through which punishments enhance reward learning.

First, we validated the reinforcement-learning model's ability for parameter estimation. The model included learning-rate parameters, which determine how quickly PEs update value, and an inverse temperature parameter, which controls the decision noise (or stochasticity of choice selection). The model—which included separate learning rates for positive and negative PEs—provided a better fit to the data than a single-learning-rate model (Fig. S2*A*). Simulations (Fig. 1*D*, white dots) and behavioral predictions (Fig. 1*F*) closely tracked observed behavior (all correlations *r* > 0.95, *p* < 0.001), and parameter recovery analyses showed reliable estimation (all correlations 0.48 < *r* < 0.96, *p* < 0.001; Fig. S2*B*).

Because learning rate and reward sensitivity can produce similar behavioral patterns, we also fitted an extended model including a reward-sensitivity parameter scaling outcome values before updating (Huys et al., 2013). This additional parameter did not improve model fit and showed poor identifiability (Fig. S2*C*,*D*). Likelihood ratio tests likewise indicated that the simpler model provided a better fit for 95–100% of participants across contexts. Although reward-sensitivity effects cannot be entirely ruled out, we therefore retained the simpler model for all analyses.

Having established model validity, we examined the impact of context on reward-learning rates for positive and negative PEs. We found a main effect of context on reward-learning rates (*F*_(1,101)_ = 4.62, *p* = 0.034, *η*^2^ = 0.010), though the interaction was not significant (*F*_(1,101)_ = 0.025, *p* = 0.53, *η*^2^ = 0). Specifically, reward-learning rates for both positive and negative PEs were higher in the reward-mixed than in the reward-only context (Fig. 1*G*). These results indicate that the presence of punishments enhances learning from both unexpected rewards (positive PEs) and their omission (negative PEs), consistent with our behavioral findings. We also confirmed that this effect was robust across model fitting approaches (Fig. S3*A*).

Given that learning rates and inverse temperature are typically correlated and both influence trial-by-trial updating, we conducted a complementary analysis to examine a composite index reflecting their joint contribution. This composite—computed as the product of learning rates and inverse temperature (Schönberg et al., 2007)—showed the same pattern, with higher values in reward-mixed than in reward-only contexts (*t*_(101)_ = 4.95, *p* < 0.001, *d* = 0.49; Fig. S3*B*), confirming that both parameters jointly contributed to behavioral differences. However, in a complementary analysis in which the inverse temperature was held constant across contexts, reward-learning rates remained significantly higher in the mixed than in the separate context (*F*_(1,101)_ = 33.40, *p* < 0.001, *η*^2^ = 0.065), suggesting that these effects are unlikely to be explained solely by variability in decision noise.

To further test whether the context effect was driven more by reward-learning rates or by inverse temperature, we compared four model variants in which these parameters were either shared or allowed to vary across contexts. All models except the fully shared-parameter model reproduced the behavioral effect (Fig. S2*E*), showing that learning rate and inverse temperature can partly compensate for each other. However, the model with context-dependent learning rate (and a shared inverse temperature) was slightly preferred (lowest BIC; Fig. S2*F*). A direct comparison of the two equal-parameter models—one varying only learning rate and one varying only inverse temperature—showed similar results. Although not decisive, variational Bayesian model selection slightly favored the context-dependent learning-rate model (protected exceedance probability = 0.55). These results suggest that the performance difference between contexts is likely better explained by changes in reward-learning rates than by changes in inverse temperature.

Parameter-recovery analyses from the original model supported this pattern. In the reward-mixed context, learning rates and inverse temperature separated more cleanly than in the reward-only context (Fig. S2*B*), indicating that the presence of punishments reduces the confounding between reward-learning rates and decision noise. Regression analyses confirmed that both parameters contributed to reward accuracy, but with asymmetric effects across contexts. In the reward-mixed context, the positive-PE learning rate remained a strong predictor of accuracy even after controlling for inverse temperature (*β* = 13.88, *p* < 0.001), whereas this relationship was not significant in the reward-only condition (*β* = 8.97, *p* = 0.051). Together, these results indicate that punishments modulate reward-related learning parameters, with learning-rate differences contributing to this effect.

Additionally, although behavioral differences in punishment trials were less pronounced, we examined whether punishment-learning rates for positive and negative PEs varied by context. A significant interaction between context and PE valence was observed (*F*_(1,101)_ = 11.29, *p* = 0.001, *η*^2^ = 0.034; Fig. 1*H*). Specifically, learning rates for negative PEs were higher in the punishment-mixed context than in the punishment-only context (*t*_(101)_ = 2.81, *p* = 0.006, *d* = 0.28). In contrast, learning rates for positive PEs did not significantly differ between contexts (*t*_(101)_ = −1.95, *p* = 0.054, *d* = −0.19). This interaction remained significant for the product between learning rates and inverse temperature (*F*_(1,101)_ = 91.04, *p* = 0.001, *η*^2^ = 0.035; Fig. S3*B*). Model comparison further favored a simple model with shared learning-rate and inverse-temperature parameters across contexts (Fig. S2*F*), consistent with the absence of robust context effects in punishment-avoidance behavior. These findings suggest that while rewards may enhance learning from unexpectedly worse punishments, they do not similarly facilitate learning from the omission of punishments, potentially contributing to lack of significant improvements in punishment avoidance within mixed contexts.

In sum, our modeling results indicate that punishments modulate reward-related learning parameters. Although learning rate, decision noise, and reward sensitivity all shape behavior in stationary tasks, convergent evidence from model comparison, parameter recovery, and accuracy regressions suggests that the context effect is driven in part by changes in reward-learning dynamics rather than by sensitivity or decision noise alone.

Together, the behavioral and modeling results suggest that punishments enhance reward learning, plausibly by modulating reward-PE signals. fMRI can capture hemodynamic responses linked to reward-PE activity in striatal regions that receive dense dopaminergic input. We therefore used fMRI in Study 2 to test whether punishments also amplify striatal reward-PE responses.

### Study 2: fMRI dataset

#### Behavioral and modeling effects do not replicate Study 1 but show convergent directional trends

In an independent fMRI study (*N* = 26), participants completed a reinforcement-learning task structurally matched to Study 1 (Fig. 1*A*,*B*; see Materials and Methods). We first tested whether the behavioral effects observed in Study 1 replicated in this independent sample.

**Figure 2.**
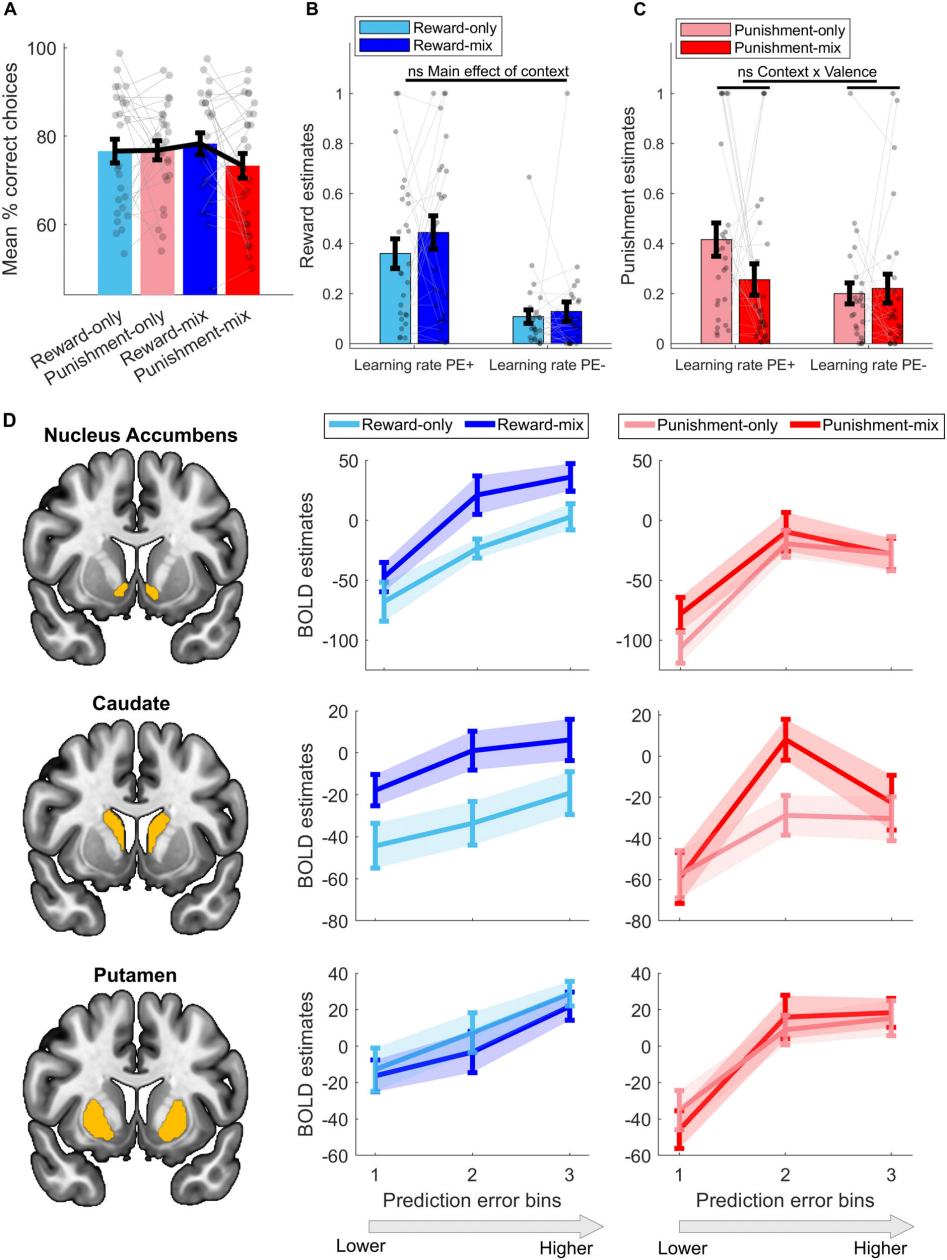
Study 2, fMRI dataset: behavioral, model fitting results and striatal BOLD responses (*N* = 26). Participants performed the reinforcement-learning task depicted in Figure 1*A*,*B* while undergoing fMRI. ***A***, Behavioral performance across contexts. Colored bars represent mean accuracy (percentage of choices for the high-probability reward or low-probability punishment stimuli), with black error bars denoting ± SEM. ***B***, ***C***, Model-estimated learning rates for reward (***B***) and punishment (***C***) trials, separately for positive (PE+) and negative (PE−) prediction errors. For reward trials, PE^+^ corresponds to better-than-expected rewards (reward delivery), and PE^−^ corresponds to worse-than-expected rewards (reward omission). For punishment trials, PE^+^ corresponds to better-than-expected punishments (punishment omission), and PE^−^ corresponds to worse-than-expected outcomes (punishment delivery). The corresponding effects from Study 1 did not reach significance in Study 2. ns, nonsignificant (*p* > 0.05). ***D***, Striatal BOLD estimates (i.e., beta coefficients) to signed PEs, grouped into three bins based on magnitude (bin 1 = lowest; bin 3 = highest), across striatal subregions: nucleus accumbens (top), caudate (middle), and putamen (bottom). Responses are shown separately for reward-only (blue, light lines), punishment-only (red, light lines), and mixed (dark lines) contexts. Error bars indicate mean ± SEM. The left panel displays the striatal masks in yellow used for BOLD signal extraction.

Specifically, we evaluated the prespecified replication contrast used for sample-size determination, derived from the Study 1 interaction: (reward-mixed − punishment-mixed) > (reward-only − punishment-only). This one-tailed test did not reach statistical significance, although the effect was numerically in the predicted direction (*t*_(25)_ = 1.44, *p* = 0.081, *d* = 0.28; Fig. 2*A*). Thus, the primary behavioral interaction observed in Study 1 was not statistically replicated in the fMRI dataset, despite showing a convergent directional trend.

To assess whether simpler effects followed the same pattern, we conducted exploratory within-context comparisons. In the mixed context, participants were significantly better at seeking rewards than avoiding punishments (*t*_(25)_ = 1.83, *p* = 0.04, *d* = 0.36, one-tailed), whereas no such difference was present in separate blocks (*t*_(25)_ = −0.08, *p* = 0.53, *d* = −0.02, one-tailed). However, this mixed-context effect did not remain significant after correction for multiple comparisons (Bonferroni-adjusted *p* = 0.08).

The absence of statistical replication may reflect limited power in this fMRI sample. Although the study was designed to achieve 80% power to detect the behavioral interaction observed in Study 1 (*d* = 0.47), prespecified exclusions reduced the final sample size, yielding ∼75% power for an effect of that magnitude. Moreover, the interaction effect observed in the fMRI dataset was substantially smaller (*d* = 0.28), corresponding to an achieved power of ∼40%, in contrast to the high power achieved in Study 1 (>96%).

Computational modeling was consistent with the behavioral results. In this fMRI sample, the main effect of mixed context on reward-learning rates did not reach statistical significance (*F*_(1,25)_ = 0.97, *p* = 0.33, *η*^2^ = 0.011). Nevertheless, the pattern of results mirrored Study 1 (Fig. 2*B*), with higher reward-learning rates—driven by both positive and negative PEs—in the reward-mixed context relative to the reward-only context. Punishment-learning rates also exhibited the same qualitative pattern as in Study 1 (Fig. 2*C*), again without reaching statistical significance (*F*_(1,25)_ = 1.18, *p* = 0.29, *η*^2^ = 0.017). Likewise, the composite measure combining learning rate and inverse temperature did not reach significance (all *p* > 0.29; Fig. S3*C*), although it followed the same directional trend as in Study 1.

In light of these results, Study 2 provides no statistical replication of the behavioral or computational effects observed in Study 1. However, the consistency in effect direction across behavioral and modeling analyses suggests a qualitatively similar pattern. These convergent patterns are compatible with the hypothesis that punishments may enhance reward learning via amplification of PE updating.

#### Punishments amplify reward-prediction error signals in the caudate

To test the hypothesis that punishments amplify reward-PE signals in the striatum, we analyzed fMRI responses in three subregions—nucleus accumbens, caudate, and putamen—which are linked to different roles in reward learning and action selection (O’Doherty et al., 2004; Yin and Knowlton, 2006; Balleine et al., 2007; Haber and Knutson, 2010).

We first examined trial-by-trial signed PEs as continuous parametric modulators of the BOLD response but found no evidence of differences in the BOLD–PE slope between reward-mixed and reward-only conditions in any of the striatal subregions (all nonsignificantly different from zero, all *p* > 0.11). However, this analysis primarily captures slope differences (i.e., the linear increase in BOLD response from negative to positive PEs) and is therefore insensitive to context effects that may manifest instead as baseline shifts. Under adaptive-coding frameworks, punishments may alter the baseline against which rewards are evaluated, effectively rescaling reward PEs.

We therefore grouped signed reward PEs into three bins and extracted BOLD estimates from each striatal subregion to assess context-dependent baseline shifts. We observed significantly stronger reward-PE signals in the reward-mixed context (which included rewards and punishments) compared with the reward-only context in the caudate (*F*_(1,25)_ = 10.45, *p* = 0.003, *η*^2^ = 0.012; Fig. 2*D*). This context effect in the caudate survived Bonferroni’s correction across the three ROIs tested (Bonferroni-adjusted *p* < 0.017). A similar effect was observed in the nucleus accumbens (*F*_(1,25)_ = 5.52, *p* = 0.027, *η*^2^ = 0.059), but it did not survive correction (Bonferroni-adjusted *p* > 0.017). No significant differences were found in the putamen (*F*_(1,25)_ = 0.79, *p* = 0.38, *η*^2^ = 0.007). A significant context × subregion interaction confirmed that the effect was region-specific (*F*_(1,25)_ = 7.75, *p* = 0.001, *η*^2^ = 0.025).

Together, these results suggest that punishments amplify the overall amplitude of striatal reward-PE responses—particularly in the caudate—by shifting the baseline of reward-PE responses rather than changing their parametric scaling.

Although our primary aim was to examine how punishments affect reward learning, we also tested the reverse: whether rewards modulate punishment-PE signals. No significant differences emerged between punishment-mixed and punishment-only contexts in any subregion (all *p* > 0.063; Fig. 2*D*). This asymmetry suggests that striatal punishment-PEs are less susceptible to contextual modulation by rewards.

**Figure 3.**
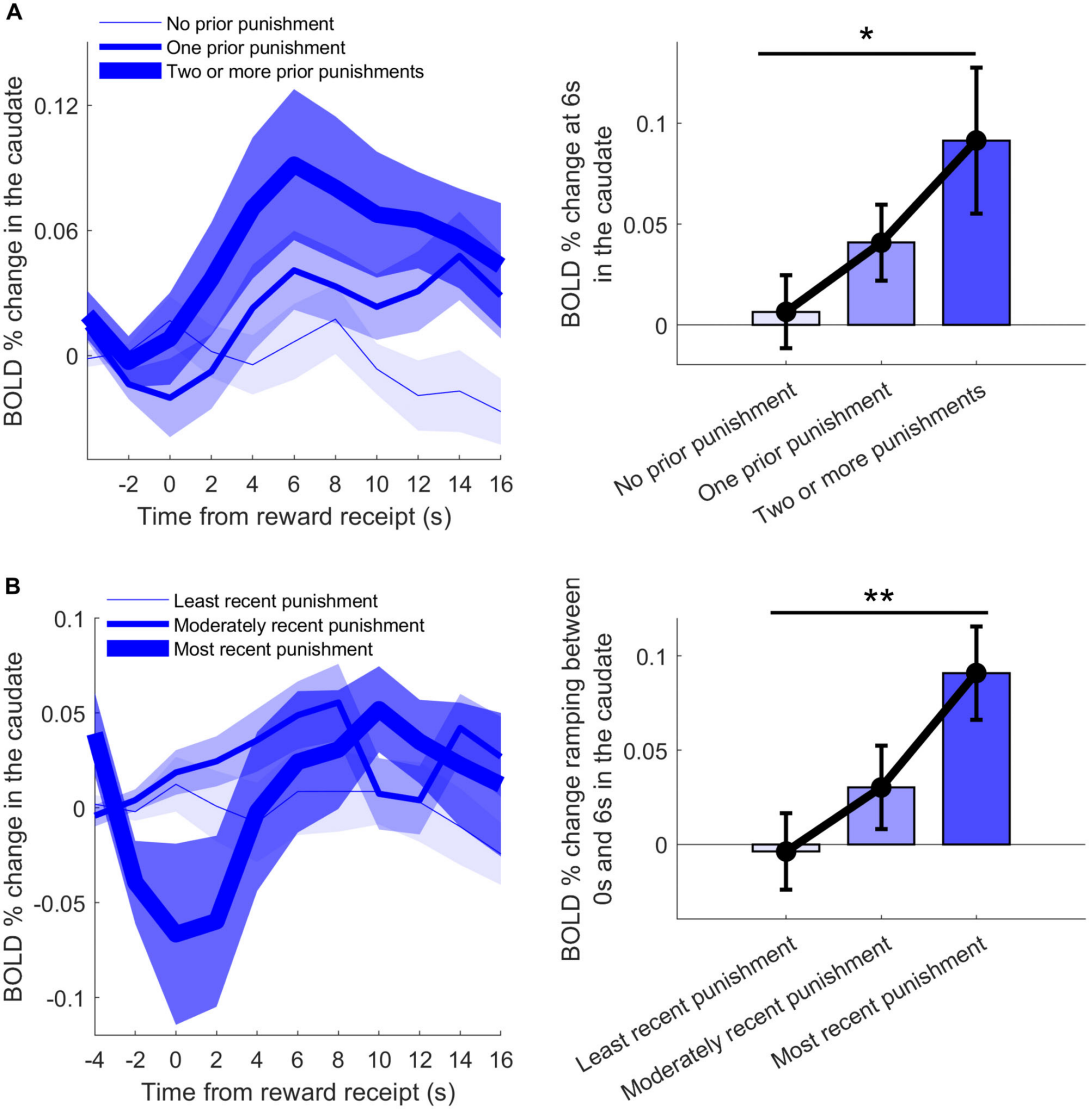
Caudate reward-receipt responses as a function of punishment history (*N* = 26). ***A***, BOLD % change in the caudate, time-locked to reward receipt, were split by the number of preceding punishments since the last reward (no prior punishment, one prior punishment, two or more prior punishments). Bar plot (right) shows the averaged BOLD signal at 6 s postreward delivery for each of the three prior punishment levels, with a linear increase across levels. ***B***, BOLD % change split by the recency of the most recent punishment (least, moderately, most recent punishment). Bar plot (right) shows ramping, defined as the averaged difference in BOLD signal between 0 s and 6 s postreward, increasing with punishment recency. *p < 0.05, **p < 0.01 refer to comparisons between the highest and lowest bins. Shaded areas (left) and bars (right) represent ± SEM.

While we showed that punishments amplify reward learning signals in the striatum, we also wanted to test whether this amplification is dynamically modulated by punishment history. To this end, we analyzed BOLD time courses as a function of the amount and recency of prior punishments. In the caudate, reward outcomes that were preceded by a greater number of punishments, evoked significantly stronger phasic responses that scaled in a parametric fashion (*t*_(25)_ = 1.85, *p* = 0.038, *d* = 0.20; Fig. 3*A*). In parallel, we found that more recent punishments were parametrically associated with a deeper initial dip in the evoked BOLD signal and stronger subsequent ramping of the reward response (*t*_(25)_ = 2.68, *p* = 0.006, *d* = 0.21; Fig. 3*B*).

These effects were specific to the caudate, with no significant modulation observed in the nucleus accumbens or putamen (all *p* > 0.05). Additionally, caudate responses to reward omission were not significantly modulated by punishment history (*p* > 0.8). This temporal profile suggests that punishment history transiently suppresses baseline striatal activity, thereby expanding the dynamic range available for subsequent reward responses (i.e., positive PEs). These results provide converging evidence that the striatum dynamically modulates reward responses as a function of prior punishment, consistent with an adaptive, context-sensitive reward learning mechanism.

Together, these fMRI results indicate that punishments dynamically modulate reward-related signaling in striatal circuits, consistent with adaptive-coding accounts often linked to dopaminergic signaling. In contrast, reward contexts do not exert comparable effects on punishment-learning signals, indicating an asymmetrical influence between the two systems.

### Study 3: fMRI meta-analysis

#### Meta-analytic evidence complements empirical findings that punishments amplify striatal reward responses

**Figure 4.**
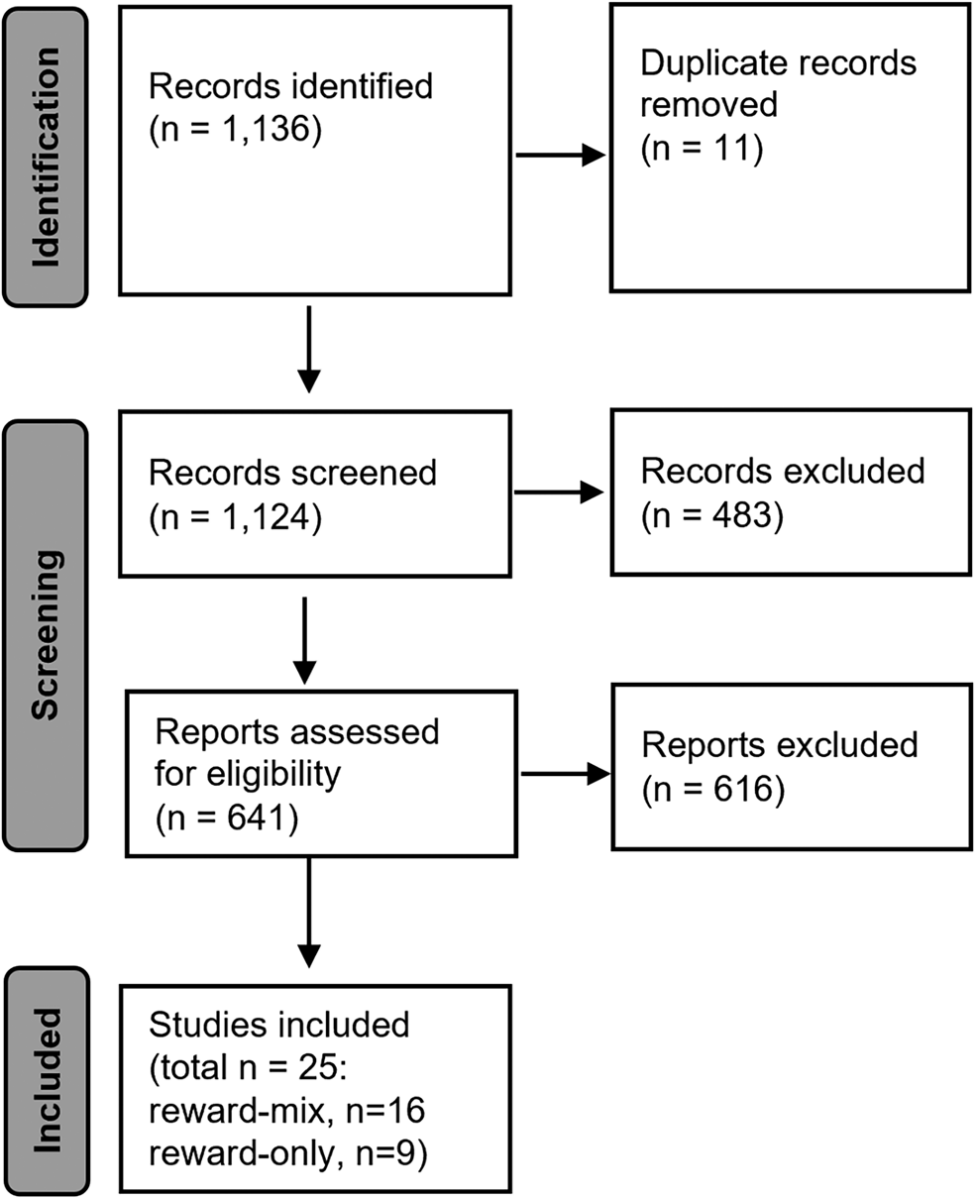
Preferred reporting items for systematic reviews and meta-analyses (PRISMA). We first identified 1,135 records through database searches in PubMed and one additional source from the authors, removed duplicates, screened 1,124 records, and assessed 641 full-text articles for eligibility. Twenty-five eligible studies of reward outcomes were included in the fMRI meta-analysis (16 for reward-mixed contexts and 9 for reward-only contexts).

To test whether the amplification of striatal reward signals by punishment contexts generalizes across the literature, we conducted an fMRI meta-analysis comparing neural responses to rewards in reward-mixed versus reward-only contexts (Fig. 4).

Most fMRI studies include either rewards or punishments (reward- or punishment-only contexts), or both (mixed contexts), but do not compare these contexts directly. Our aim was to assess whether prior findings align with the pattern observed in our empirical neural data.

We restricted inclusion to categorical reward receipt contrasts (reward > no reward) rather than parametric PE regressors. This decision was based on our own findings: context primarily modulated the overall magnitude of PE responses rather than their linear slope (Fig. 2*D*), making categorical comparisons more appropriate. Our meta-analysis thus targeted reward outcomes corresponding predominantly to our higher PEs bin (bin 3) at the time of reward receipt (see Fig. S4*A* for the overlap between higher PEs bin and reward > no reward contrasts in our empirical data). We identified 25 studies using either reward-mixed or reward-only designs and suitable contrasts (Table S1). After applying the exclusion criteria, all included studies used only secondary punishments in mixed contexts consistent with our own design (studies using primary punishments are more widely used in punishment-only conditioning paradigms; Corlett et al., 2022). Due to limited data (*n* = 4), we were unable to analyze reward omission contrasts reliably and thus focused only on reward receipt.

**Figure 5.**
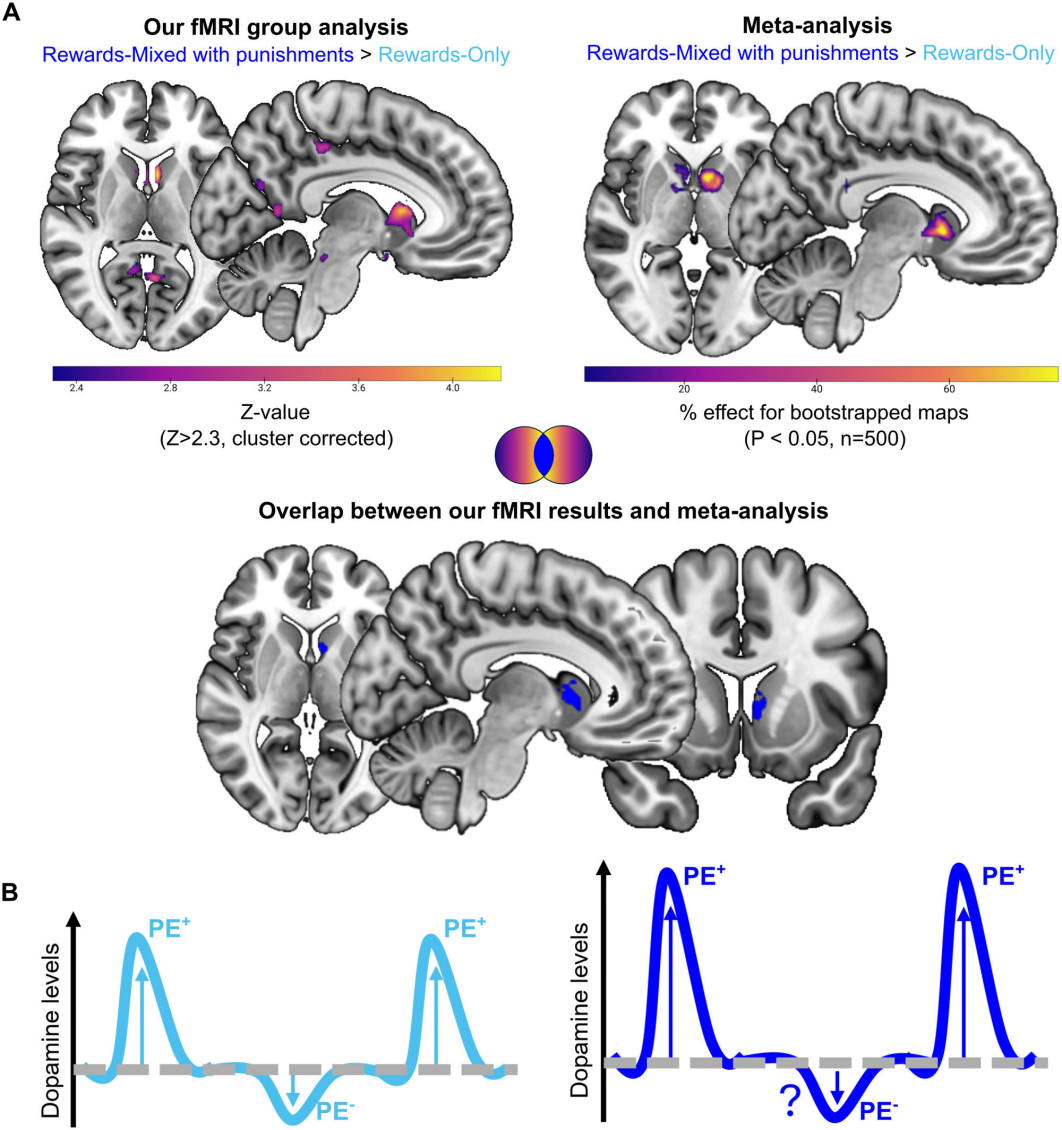
Study 3, meta-analysis comparison with empirical fMRI results and proposed neurobiological model. ***A***, Brain maps comparing empirical and meta-analytic results for the contrast reward-mixed > reward-only. The left panel shows empirical fMRI activation maps for higher prediction errors (PEs, bin 3), while the right panel displays meta-analytic activation maps derived from 500 bootstrap iterations. Warmer colors (yellow) indicate stronger effects. Notably, overlapping activations were observed in the caudate (cluster in solid blue), suggesting consistency between our empirical findings and prior literature. ***B***, Schematic representation of the proposed neurobiological framework for the effect of punishments on reward PEs. The presence of punishments (dark blue, right) might amplify dopamine-related signals for positive prediction errors (PE^+^, upward arrows), potentially enhancing reward learning relative to reward-only contexts (light blue, left). Further research is needed to clarify the specific neurobiological mechanisms influencing negative prediction errors (PE^−^, downward arrows).

The meta-analysis results aligned with our empirical fMRI data. We found stronger striatal reward-related activity in reward-mixed contexts compared with reward-only (Fig. 5*A*). Crucially, this effect was mostly localized to the caudate—overlapping with 41% of the striatal voxels identified in our empirical fMRI study. No clusters emerged in the reverse contrast (reward-only > reward-mixed), suggesting the observed striatal enhancement is primarily driven by the presence of punishments. Additionally, we further confirmed that the effect held even when including and classifying studies with correct/incorrect feedback as either reward-only or reward-mixed contexts (Fig. S4*B*, Table S1), indicating that our result was robust to classification approach.

Overall, these meta-analytic findings reinforce our empirical results: across independent datasets, reward-related striatal activity is consistently stronger in the presence of punishments. This convergence points to a systematic contextual modulation of reward learning by punishment, highlighting the importance of considering the broader outcome context when interpreting reward-related signals.

## Discussion

Our study shows that punishments sharpen reward learning by amplifying striatal PE signals, particularly in caudate. This effect was consistently observed across behavioral, computational, neuroimaging, and meta-analytic evidence. In real environments rewards and punishments frequently co-occur, yet most research investigates them separately without considering potential influences over each other. Our data challenge this separation: when both outcomes occur in the same context, punishments increase the behavioral and neural impact of rewards. This amplification reflects a contextual upregulation of reward-PE signals in striatal circuits.

Work in nonhuman animals already points to such cross-valence interactions. Conditioned suppression (Estes and Skinner, 1941) and electrophysiological studies (Matsumoto and Hikosaka, 2009; Fiorillo et al., 2013; Matsumoto et al., 2016) show that punishments modulate reward-seeking behavior and dopaminergic activity in reward-related regions. Our results extend this to humans, demonstrating that the same kind of behavioral and neural modulation operates during concurrent reward–punishment learning.

Importantly, our results speak specifically to contextual effects between reward and punishment histories, not to how rewards and punishments are combined within individual choices. While many economic models assume a single value dimension, other evidence suggests that reward and punishment are learned on distinct streams before being integrated for choice, supporting multi-attribute reinforcement-learning frameworks (Louie et al., 2015). We follow the latter architecture by modeling reward and punishment learning as separate streams, allowing us to isolate how outcome context shapes each pathway. Although we do not model their integration at the choice level, our findings show that the mere presence of punishments can recalibrate how the brain responds to reward outcomes. This aligns with prospect theory and adaptive/relative coding accounts in which recent negative events shift the reference point used to evaluate subsequent rewards (Vlaev et al., 2011).

Consistent with this interpretation, punishment effects on reward learning appear largely additive rather than multiplicative: reward learning curves remained largely parallel across contexts, learning rates were modulated similarly for positive and negative PEs, and striatal reward-PE showed increased response amplitude (consistent with baseline shift) despite unchanged slope. This additive pattern aligns with adaptive-coding accounts in which punishments may shift the reference baseline for reward encoding.

These results also help resolve other conflicting findings in the literature. Some studies report that ongoing painful stimulation heightens reward responsivity (Gandhi et al., 2013; Wang et al., 2019), while others show that rewards are suppressed when reward and pain outcomes stem from the same decision (Talmi et al., 2009). Our data can explain this divergence: punishments enhance reward learning when the two outcomes co-occur in the same context but arise from separate, not the same, decisions—a key distinction that has often been overlooked.

Comparing mixed versus separate outcome contexts suggests that reward-only paradigms may underestimate reward responses. When rewards and punishments occur together—closer to real-world environments—striatal reward signals are larger, consistent with enhanced reward learning. Our meta-analysis converges on this pattern: studies that include both outcome types show stronger reward-related activity than reward-only tasks. These findings indicate that mixed-valence contexts—common in both laboratory and real-world learning—systematically shape how reward signals are expressed in the brain. Accordingly, future work should consider outcome context when interpreting striatal responses to rewards.

Unlike prior work using primary punishments (e.g., painful shocks; Talmi et al., 2009; Gandhi et al., 2013; Wang et al., 2019), we employed monetary losses, keeping reward and punishment within the same sensory domain. Behavioral and fMRI data nonetheless showed effective avoidance learning, indicating that these losses were motivationally relevant. Primary punishments strongly engage salience systems and can elicit dopaminergic responses (Seymour et al., 2007; Matsumoto and Hikosaka, 2009; Fiorillo et al., 2013), whereas monetary losses more reliably suppress striatal activity (Delgado et al., 2000; Tom et al., 2007; Cooper and Knutson, 2008); nevertheless, both may amplify reward PEs via distinct mechanisms.

Importantly, our findings point to a potential asymmetry, whereby punishments enhanced reward learning, whereas rewards did not similarly enhance punishment learning. This pattern is consistent with a directional modulation account rather than a reciprocal one. It contradicts models proposing either fully integrated and symmetric valence systems or independent mechanisms. Although we cannot fully exclude separable pathways for reward and punishment learning (Palminteri and Pessiglione, 2017), our results support an architecture in which the two signals are integrated in shared regions (Seymour et al., 2012) but modulate each other asymmetrically. Functionally, such directional modulation may be adaptive, as increasing reward-seeking in the presence of punishment could facilitate recovery from negative outcomes.

The observed asymmetry is difficult to reconcile with simple arousal-based accounts, which would predict a global increase across both reward and punishment signals. A more plausible, but still speculative, explanation is a form of adaptive coding—where the dopaminergic system rescales PE signals based on recent outcomes. Under this framework, punishments can transiently lower baseline dopamine and expand the dynamic range for subsequent reward PEs (Leknes and Tracey, 2008; Gee et al., 2020), or they can shift the effective coding range based on outcome distribution (Tobler et al., 2005). Both mechanisms could predict stronger reward responses in punishment-rich contexts and fit our results better than a global arousal explanation. Still, without direct dopaminergic measurements, our data cannot distinguish between these putative mechanisms, so we interpret the effect as a contextual recalibration of reward learning rather than evidence for a specific dopaminergic mechanism.

Nonetheless, our BOLD findings lend some support to the adaptive-coding interpretation. Reward-evoked caudate activity scaled with recent punishment history: rewards following more punishments elicited larger responses, and recent punishments induced a deeper early dip followed by a sharper rise in the reward response. This pattern fits the idea that punishments transiently suppress striatal baseline activity, widening the dynamic range for subsequent reward signals. Crucially, we found no comparable history effects on reward omissions, ruling out simple hemodynamic carryover from the previous punishment and aligning with evidence that negative PEs are less sensitive to dopaminergic modulation (Pessiglione et al., 2006). Future work will be needed to determine how these BOLD shifts reflect dopaminergic dynamics.

Although the most robust effect of punishment was the dynamic amplification of positive reward PEs—the primary driver of reward learning in standard reinforcement models—we also observed stronger negative PEs (i.e., signals following unexpected reward omission). The functional significance of this effect is less clear (Fig. 5*B*), and the direction of the BOLD response suggests downstream processes (Ullsperger and von Cramon, 2004) beyond canonical dopaminergic dips. Alternatively, these effects could reflect the influence of other neuromodulators such as serotonin or noradrenaline (Aston-Jones and Cohen, 2005; Cools et al., 2011), although direct evidence in mixed contexts is limited.

The enhancement of reward-PE signals in the nucleus accumbens and caudate fits established functional roles of these regions in representing value and PEs (O’Doherty et al., 2004; Seymour et al., 2004; Fouragnan et al., 2018). The putamen showed no such modulation, consistent with its stronger involvement in motor and habitual processes (Yin and Knowlton, 2006; Balleine et al., 2007). This suggests that the punishment-induced amplification primarily reflects value-specific processing in regions specialized for outcome evaluation, rather than a generalized striatal response.

Our results carry implications for computational modeling. Traditional reinforcement-learning frameworks typically analyze reward and punishment learning as independent processes. However, our data suggest an interplay between these two systems, with reward-learning rates and reward-PEs modulated by punishments. In this study, we deliberately employed well-validated, standard reinforcement-learning models (Pessiglione et al., 2006; Frank et al., 2007) as a starting point for identifying robust neural correlates of PEs in striatal circuits, thereby providing a principled framework for assessing these contextual effects on each learning stream. Nonetheless, our results indicate that reward and punishment computations may interact more dynamically than these standard models assume. We view our findings as an initial benchmark against which to evaluate richer computational accounts. In particular, future models should test adaptive-coding mechanisms in which punishment outcomes dynamically alter parameters governing reward learning.

Several limitations should be noted. The behavioral and modeling effects did not statistically replicate in the smaller fMRI sample, likely due to reduced power, increased task difficulty and scanner environment (Charpentier et al., 2021). Accordingly, these results should be interpreted as convergent rather than confirmatory. Moreover, the stationary task design limits dissociation of learning rates from other computational parameters, which future studies using dynamic environments should address. Additionally, individual differences in loss aversion may contribute to the contextual effects (Tom et al., 2007), but our key finding—selective enhancement of reward learning rather than a general effect—are unlikely explained by loss aversion alone.

In conclusion, our findings demonstrate that punishment contexts sharpen reward learning by amplifying striatal PE signals. These results challenge standard models that treat reward and punishment learning as separable processes and highlight the critical role of contextual interdependence between these two systems. Beyond advancing theoretical understanding, these insights may shed light on real-world phenomena and mental health—such as why negative events can paradoxically enhance reward-seeking behavior, or why drug relapse often follows negative experiences—and point to new opportunities for designing behavioral interventions that harness contextual modulation of reward learning.

## Data Availability

All data needed to evaluate the conclusions in the paper are present in the paper and/or the supplementary information. Raw data will be made available upon request to the corresponding authors.
